# Prognostic significance of the CRP–albumin–lymphocyte (CALLY) index in esophageal cancer: systematic review and meta-analysis

**DOI:** 10.7717/peerj.21277

**Published:** 2026-05-22

**Authors:** Wen Xu, Daqin Zhan, Ruolan Wang, Shu Jin, Xiao-Bo Liu

**Affiliations:** 1 Department of Gastroenterology, Taihe Hospital, Hubei University of Medicine, Shiyan, Hubei, China; 2 Department of Pediatrics, Taihe Hospital, Hubei University of Medicine, Shiyan, Hubei, China

**Keywords:** Esophageal cancer, CRP–albumin–lymphocyte index, Meta-analysis

## Abstract

**Background and Objective:**

Esophageal cancer (EC) is associated with marked regional disparities, poor 5-year survival (15%–25%), and limited progress in therapeutic outcomes. The tumor, node, and metastasis (TNM) staging system has reduced accuracy for long-term survival prediction, particularly after neoadjuvant therapy. The C-reactive protein–albumin–lymphocyte (CALLY) index—integrating inflammation (C-reactive protein), nutrition (albumin), and immunity (lymphocytes)—emerges as a novel prognostic biomarker. This study aims to evaluate its association with survival outcomes in patients with EC.

**Methods:**

Following the PRISMA guidelines and PROSPERO registration (CRD420251125031), two researchers independently screened and extracted data from all relevant original articles published from database inception to July 2025. Study quality was assessed using the Newcastle-Ottawa Scale (NOS) score. Meta-analysis was performed using RevMan 5.3 software, and the risk of bias was assessed using risk of bias plots and funnel plots.

**Results:**

Seven studies involving 2,243 patients were included in this meta-analysis. Pooled multivariate analyses of the included studies demonstrated that the CALLY index was independently associated with 5-year overall survival (OS) and recurrence-free survival (RFS), with a high CALLY index consistently predicting favorable outcomes. Moreover, patients with EC in the high CALLY index group had better 5-year OS and RFS than those in the low CALLY index group.

**Conclusion:**

The pretreatment CALLY index is a robust prognostic biomarker that is independently associated with improved OS and RFS in patients with EC.

## Introduction

Esophageal cancer (EC) remains one of the most common fatal malignancies worldwide, with a 5-year overall survival rate of 15% to 25% (Ma et al., 2024). Its incidence and mortality exhibit marked regional disparities, and its prevalence is influenced by specific environmental and lifestyle factors. Approximately 75% of deaths due to EC occur in Asia, with China being a high-incidence area characterized by a distinct “esophageal cancer high-risk belt” region (Bray et al., 2024; Han et al., 2024). EC is managed with multimodal therapy, including chemotherapy, radiotherapy, surgery, and immunotherapy. However, therapeutic progress has been slow and incremental, with no significant improvement in survival rates observed over the past 5–10 years (Han et al., 2024). Therefore, highly efficient and sensitive predictive tools are urgently needed to enable the precise risk stratification for patients with EC and guide clinical decision-making, thereby improving patient prognosis.

The tumor, node, and metastasis (TNM) staging system is widely used for risk stratification and treatment planning in EC (Rice et al., 2017). Nevertheless, it has limitations and a significantly reduced predictive accuracy for the long-term survival of patients with EC receiving neoadjuvant chemoradiotherapy (Rizk et al., 2007). Furthermore, significant survival heterogeneity persists among patients within the same TNM stage (Rizk et al., 2007). These observations indicate the limited predictive capacity of TNM staging for the complex biological behavior of EC. In recent years, inflammation-, immune-, and malnutrition-related indices have been increasingly used to assess cancer prognosis. Numerous markers, such as the neutrophil-to-lymphocyte ratio (NLR), lymphocyte-to-monocyte ratio, platelet-to-lymphocyte ratio (PLR), C-reactive protein (CRP), modified Glasgow prognostic score (mGPS), systemic immune–inflammation index (SII), and systemic inflammation response index, have been proven to be significantly associated with the prognosis of various gastrointestinal cancers (Wu et al., 2025). A composite index named CRP–albumin–lymphocyte (CALLY) has been proposed and demonstrated to be significantly associated with the prognosis of gastric cancer, colorectal cancer, and nonsmall cell lung cancer (Miyazaki et al., 2024; Wu et al., 2025). This index integrates inflammatory status (CRP), nutritional status (albumin), and immune function (lymphocytes) into a single measure, overcoming the limitations of individual markers, providing a comprehensive reflection of the overall health status of patients with cancer, and thereby offering an integrated perspective on prognosis.

Recent studies have explored the relationship between the CALLY index and the prognosis of patients with EC, compared its prognostic value with that of other classic indices, and developed a novel EC staging system based on the CALLY index, offering new insights for personalized EC treatment (Jia et al., 2025; Ma et al., 2024; Meng et al., 2025). In the present work, we conducted the first systematic review and meta-analysis to investigate the association between the CALLY index and the prognosis of patients with EC.

## Materials and Methods

### Literature search strategy

This systematic review and meta-analysis was conducted in accordance with the PRISMA guidelines (Page et al., 2021) and began with protocol submission on PROSPERO (CRD420251125031). Comprehensive computerized searches were performed on PubMed, Web of Science, Medline, Embase, and China National Knowledge Infrastructure. Google Scholar was utilized for supplemental searches. The search covered the period from database inception to July 2025 and was performed by employing a combination of Medical Subject Headings terms and free-text keywords. The main search strategy was as follows: ①. (“C-reactive protein-albumin-lymphocyte index” OR “CRP-albumin-lymphocyte index”); ②. CALLY index; ③. (“esophageal” OR “esophagus” OR “oesophageal” OR “oesophagus”); ④. (“neoplasm” OR “cancer” OR “carcinoma” OR “tumors”); ⑤. ① OR ②; ⑥. ③ AND ④; and ⑦. ⑤ AND ⑥.

### Inclusion and exclusion criteria

The inclusion criteria were ① study type: prospective or retrospective cohort studies, and other papers providing key outcome data; ② study subjects: human studies involving patients with a clinical diagnosis of esophageal cancer and assessing the prognostic ability of CALLY index; ③ the CALLY index was calculated using the following formula: preoperative serum albumin level (g/dl) × lymphocyte count (cells/µl)/C-reactive protein (mg/dl) × 10^4^. We explicitly confirmed that all included studies applied this compatible formula or performed appropriate unit conversions to ensure mathematical equivalence. This verification ensures that the index values and subsequent thresholds across studies are based on the same core calculation; ④ intervention: the patients were divided into a high CALLY index group and low CALLY index group; and ⑤ at least one of the following outcome measures: 5-year overall survival (OS), 5-year overall recurrence-free survival (RFS), and comparison of clinical characteristics between the high and low CALLY groups.

The exclusion criteria were as follows: animal studies, single case reports, reviews, commentaries, conference abstracts, and any study that lacked comparative original data between a high CALLY index group and a low CALLY index group.

All included studies assessed the pretreatment CALLY index, calculated from blood samples obtained before the initiation of any anticancer therapy (*e.g.*, chemotherapy, radiotherapy or surgery). Studies reporting a post-treatment CALLY index or evaluating dynamic changes were not included, as our primary aim was to evaluate the prognostic value of the host’s pre-therapeutic inflammatory-nutritional-immunological status.

### Data extraction and quality assessment

Literature screening was performed independently by two investigators (Zhan and Jin) following a predefined protocol registered in PROSPERO (CRD420251125031). In brief, the two reviewers screened titles, abstracts, and full texts against the eligibility criteria. Data from included studies were extracted using a standardized data collection form, which was piloted beforehand. Any disagreements during the screening or extraction process were resolved through discussion or, if necessary, adjudication by a third senior reviewer (Xu). The extracted information encompassed study design, authors, country, sample size, study period, initial treatment, TNM stage, histological type, cutoff values, OS, RFS, lymph node metastasis, vessel invasion, and postoperative complication. Methodological quality of the included retrospective cohort studies was appraised using the Newcastle-Ottawa Scale (NOS) (Stang, 2010), with studies scoring ≥6 considered high quality. The assessment was conducted independently by the same two reviewers, and the results were cross-checked.

### Statistical analysis

RevMan 5.3 software was used for data synthesis and analysis. Heterogeneity among the studies was assessed using the I^2^ statistic, which quantifies the proportion of total variation attributable to between-study heterogeneity. I^2^ values of 25%, 50%, and 75% were interpreted as low, moderate, and high heterogeneity, respectively (Higgins et al., 2024). A fixed-effects model was employed when heterogeneity was insignificant (I^2^ <50%) and studies were considered methodologically and clinically homogeneous (Higgins et al., 2024). A random-effects model was applied when I^2^ ≥ 50% or when substantial clinical or methodological diversity existed across studies, as this model provides more conservative estimates by incorporating between-study variance (Higgins et al., 2024). In the meta-analysis of 5-year OS and 5-year RFS rates, we calculated risk ratios (RRs) and 95% confidence intervals based on the number of events at 5 years in each group, using the Mantel-Haenszel method with a fixed-effects or random-effects model as appropriate. For the meta-analysis of univariate and multivariate prognostic effects, we extracted hazard ratios (HRs) and their 95% confidence intervals from Cox regression models reported in the included studies. These HRs were pooled using the generic inverse-variance method. All analyses were performed using RevMan 5.3 software.

Separate meta-analyses were conducted for univariate and multivariate prognostic effects. Univariate meta-analyses pooled HRs derived from unadjusted Cox regression models, reflecting the crude association between the CALLY index and survival. Multivariate meta-analyses pooled HRs adjusted for potential confounders such as age, sex, TNM stage, and treatment modality. These analyses were performed independently to evaluate the independent prognostic value of the CALLY index and to assess the stability of its effect across different levels of statistical adjustment. Risk of bias assessment was evaluated using a risk of bias plot, and publication bias was evaluated with a funnel plot.

## Literature Screening Process and Results (Fig. 1)

**Figure 1:**
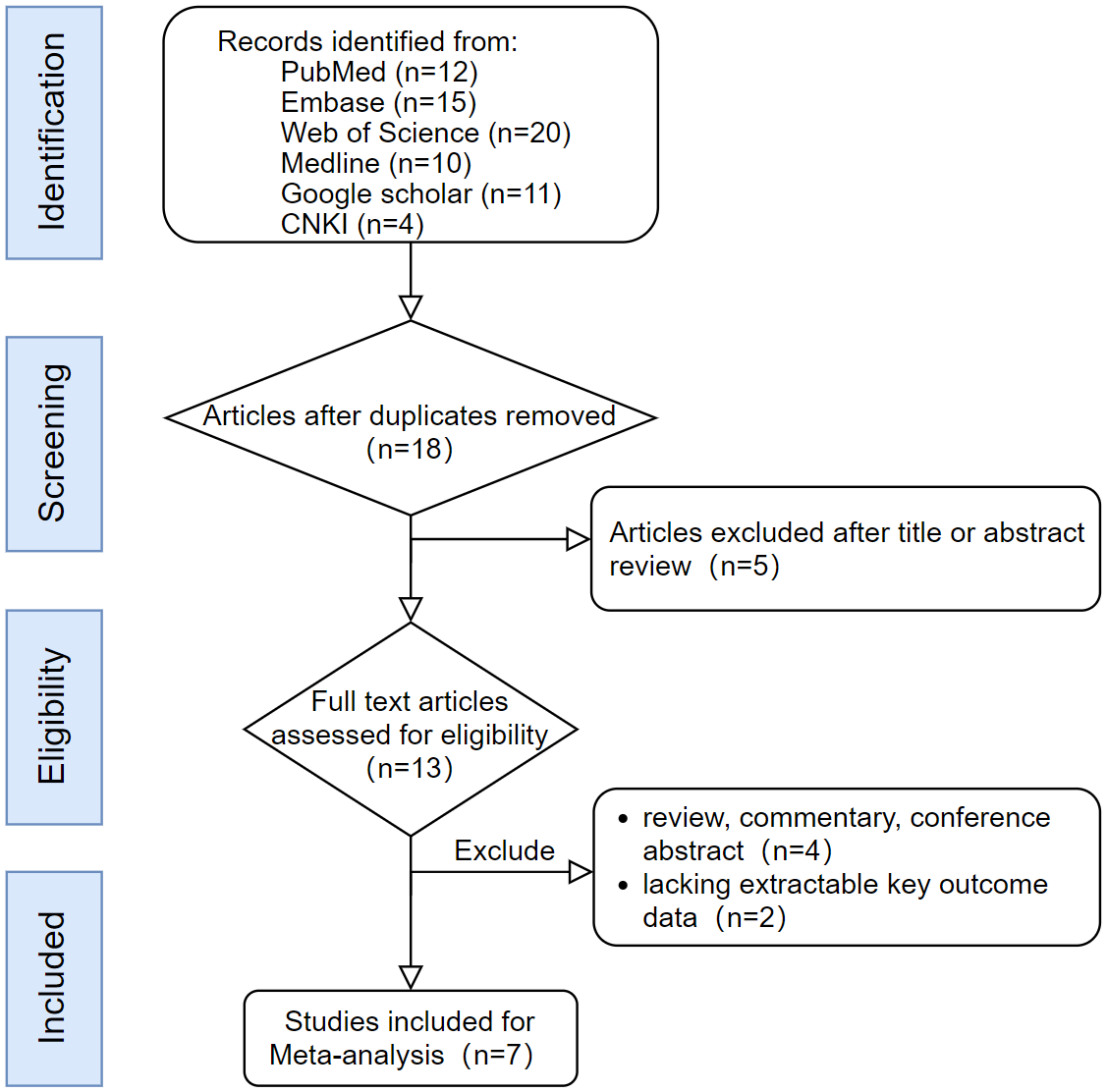
Flow diagram depicting the strategies of the meta analyses.

Seventy-two articles were retrieved initially using the search strategy. After the duplicates were removed, eighteen records remained. Screening the titles and abstracts excluded five records, leaving thirteen studies with full texts for eligibility assessment. Six articles were excluded for not meeting the inclusion criteria. Finally, seven studies (Aoyama et al., 2024; Fan et al., 2025; Feng et al., 2024; Jia et al., 2025; Ma et al., 2024; Meng et al., 2025; Xu et al., 2025) were included in the meta-analysis as depicted in the flow chart in Fig. 1. No case of missing data requiring clarification from the corresponding authors was encountered.

## Characteristics of Studies

The characteristics and NOS scores of the included studies are summarized in Table 1. Although randomized controlled trials (RCTs) were initially considered eligible, our systematic search did not identify any RCTs that investigated the prognostic value of the pretreatment CALLY index in esophageal cancer. Therefore, all seven studies included in the analysis were retrospective, with two being multicenter (Jia et al., 2025; Meng et al., 2025) and the others (Aoyama et al., 2024; Fan et al., 2025; Feng et al., 2024; Ma et al., 2024; Xu et al., 2025) being single-center. They were conducted in Japan and China, published between 2024 and 2025, and included 2,243 patients, among whom 1,169 were in the high CALLY index group and 1,074 were in the low CALLY index group. The quality of each study was assessed by the NOS and ranged from six to seven points, suggesting that all the studies were of high quality.

**Table 1:** Characteristics of the included studies.

Author (year)	Country	Study design	Sample size	Study period	Initial treatment	TNM	Histological type	Cutoff value	Method to determine cutoff	NOS score	
Ma et al. (2024)	Japan	Retro+S	146	2008–2018	Neoadjuvant chemotherapy+surgery+chemoradiotherapy	I+II 104 III+IV 42	SCC and others	^a^OS: 2.40 DFS: 4.56 SSI: 12.08	ROC	6	
Feng et al. (2024)	China	Retro+S	318	2013–2015	Surgery+ Chemoradiotherapy	I 110 II 93 III 115	SCC	1.7	ROC	6	
Meng et al. (2025)	China	Retro+M	553+104	2010–2024	Neoadjuvant chemotherapy+surgery+radiotherapy	I 98 II 275 III 134 IV 46	SCC and others	2.55	ROC	7	
Jia et al. (2025)	China	Retro+M	518	2013–2023	Surgery or other anti-tumor treatment	I 40 II 133 III 146 IV 199	SCC and others	2.51	Maximum rank statistics	7	
Aoyama et al. (2024)	Japan	Retro+S	180	2005–2020	Neoadjuvant chemotherapy+surgery (R0 resection)	I B - III	ADC and SCC	5	Based on prior reports and 3-/5-year survival rates	6	
Xu et al. (2025)	China	Retro+S	209	2019–2022	Surgery (R0 resection)	I 50 II 117 III 42	SCC	3.47	ROC	6	
Fan et al. (2025)	China	Retro+S	215	2020–2022	Surgery (R0 resection)	I 118 II 39 III 58	SCC	1.97	ROC	6	

**Notes.**

Retro Retrospective S single center M multicenter SCC squamous cell carcinoma ADC adenocarcinoma OS overall survival DFS disease-free survival SSI surgical site infection ROC receiver operating characteristics

a The study by Ma et al. (2024) determined distinct optimal CALLY index cut-off values for different clinical endpoints using ROC curve analysis: 2.40 for OS, 4.56 for DFS, and 12.08 for SSI.

## Meta-Analysis Results

### Comparison of 5-Year OS rates

**Figure 2:**
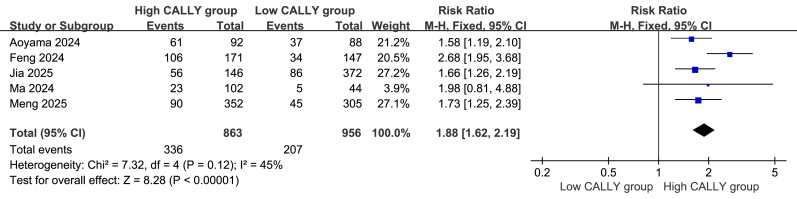
Comparison of the patients’ 5-year overall survival(OS) rate.

Five studies (Aoyama et al., 2024; Fan et al., 2025; Feng et al., 2024; Jia et al., 2025; Ma et al., 2024; Meng et al., 2025; Xu et al., 2025) were included in the meta-analysis of the 5-year OS rate. The forest plot revealed that the high CALLY index group had a higher survival rate than the low CALLY index group (RR = 1.88, 95% CI [1.62–2.19], *P* < 0.00001; Fig. 2). Homogeneity was good (*P* = 0.12, I^2^ = 45%), supporting the use of a fixed-effects model.

### Comparison of the 5-year RFS rates

**Figure 3:**
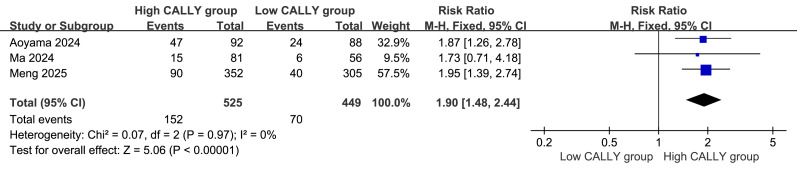
Comparison of the patients’ 5-year recurrence-free survival (RFS) rate.

Three studies (Aoyama et al., 2024; Ma et al., 2024; Meng et al., 2025) were included in the meta-analysis of the 5-year RFS rate. Homogeneity was good (*P* = 0.97, I^2^=0%), supporting the use of a fixed-effects model. The forest plot (Fig. 3) showed a higher 5-year RFS rate in the high CALLY index group compared with that in the low CALLY index group (RR = 1.90, 95% CI [1.48–2.44], *P* < 0.00001).

### Univariate and multivariate meta-analyses of OS

**Figure 4:**
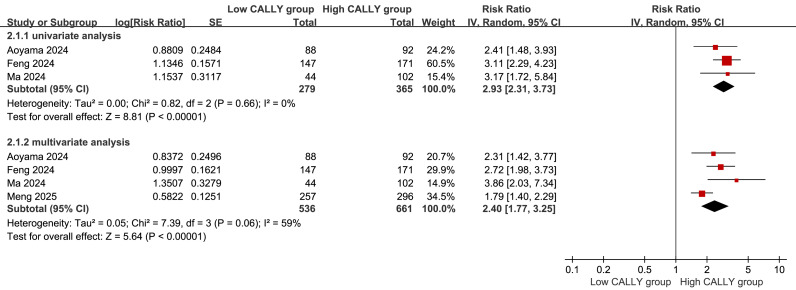
The univariate and multivariate meta-analyses of OS.

With the high CALLY index group as the control group and the low CALLY index group as the experimental group, the relationship between the CALLY index and the prognosis of patients with EC was analyzed. Three studies (Aoyama et al., 2024; Feng et al., 2024; Ma et al., 2024) and 644 patients were included in the univariate meta-analyses of OS, and four studies (Aoyama et al., 2024; Feng et al., 2024; Ma et al., 2024; Meng et al., 2025) and 1,197 patients were included in the multivariate analyses. The results of both univariate (RR = 2.93, 95% CI [2.31–3.73], *P* < 0.00001) and multivariate meta-analyses (RR = 2.40, 95% CI [1.77–3.25], *P* < 0.00001) indicated that the CALLY index was independently associated with OS, with a high CALLY index conferring a favorable prognosis (Fig. 4).

### Univariate and multivariate meta-analyses of RFS

**Figure 5:**
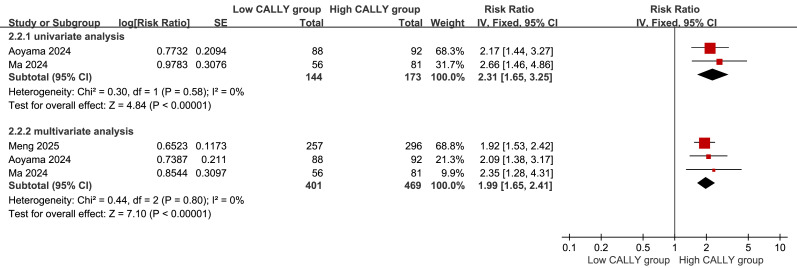
The univariate and multivariate meta-analyses of RFS.

The correlation between the CALLY index and the prognosis of patients with EC was examined using the high CALLY index group as the control group and the low CALLY index group as the experimental group. The univariate meta-analysis also included 317 patients and two studies (Aoyama et al., 2024; Ma et al., 2024), and the multivariate analyses included 870 patients and three studies (Aoyama et al., 2024; Ma et al., 2024; Meng et al., 2025). The results of univariate (RR = 2.31, 95% CI [1.65–3.25], *P* < 0.00001) and multivariate (RR = 1.99, 95% CI [1.65–2.41], *P* < 0.00001) analyses validated that the CALLY index was independently associated with RFS. Furthermore, a high CALLY index was associated with a favorable prognosis for patients with EC (Fig. 5).

### Comparison of clinical characteristics between the high and low CALLY index groups

Eight characteristics were included in this meta-analysis, and the results are shown in Table 2. Significant differences were observed in sex, T status, vessel invasion, and TNM stage. The patients with a high CALLY index were males (OR = 0.75, 95% CI [0.60–0.95], *P* = 0.01) and had earlier T status (OR = 1.73, 95% CI [1.31–2.28], *P* = 0.0003), lower vessel invasion (OR = 0.73, 95% CI [0.57–0.93], *P* = 0.03), and earlier TNM stages (OR = 1.61, 95% CI [1.20–2.17], *P* = 0.0006). No significant difference in tumor differentiation (OR = 1.08, 95% CI [0.81–1.43], *P* = 0.60), undertaking neoadjuvant therapy (OR = 1.45, 95% CI [0.97–2.16], *P* = 0.07), lymph node metastasis (OR = 0.85, 95% CI [0.67–1.08], *P* = 0.08), and postoperative complication (OR = 2.81, 95% CI [0.30–26.29], *P* = 0.36) were found between the high and low CALLY index groups. Detailed figures are provided as Figs. S1–S8.

**Table 2:** Meta-analysis of the clinical characteristics of the high CALLY group *vs.* the low CALLY group.

Characteristics	No. of study	H (n=)	L (n=)	Heterogeneity (I^2^ %)	Modle	Meta-analysis		
						OR	95% CI	*P* value
Sex (Male/Female)	6	1,064	1,033	0	Fixed	0.75	0.60–0.95	0.01
Differentiation (Well/Not well)	2	523	452	0	Fixed	1.08	0.81–1.43	0.60
Neoadjuvant therapy (Yes/No)	2	444	393	0	Fixed	1.45	0.97–2.16	0.07
T status (T1/Not T1)	4	750	511	0	Fixed	1.73	1.31–2.28	0.0003
Lymph node metastasis(P /N)	4	750	511	12	Fixed	0.85	0.67–1.08	0.08
Vessel invasion (P /N)	4	773	522	0	Fixed	0.73	0.57–0.93	0.03
TNM stage (I/Not I)	4	624	636	0	Fixed	1.61	1.20–2.17	0.0006
Postoperative complication (Yes/No)	2	239	459	95	Random	2.81	0.30–26.29	0.36

**Notes.**

H High CALLY group L Low CALLY group n Number of patients P positive N negative D Differentiated U Undifferentiated SCC Squamous cell carcinoma

### Evaluation of publication bias

**Figure 6:**
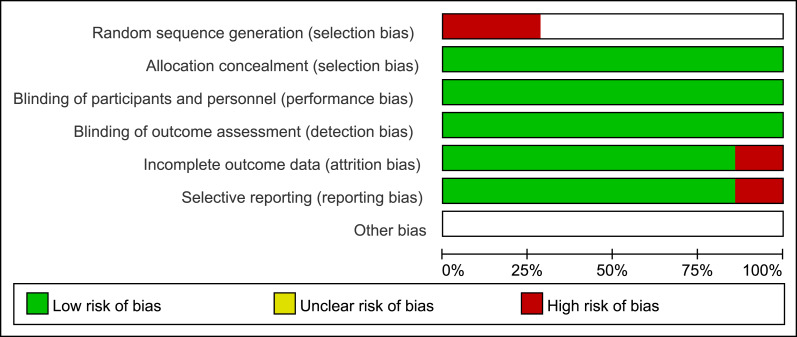
Risk of bias assessment plot.

**Figure 7:**
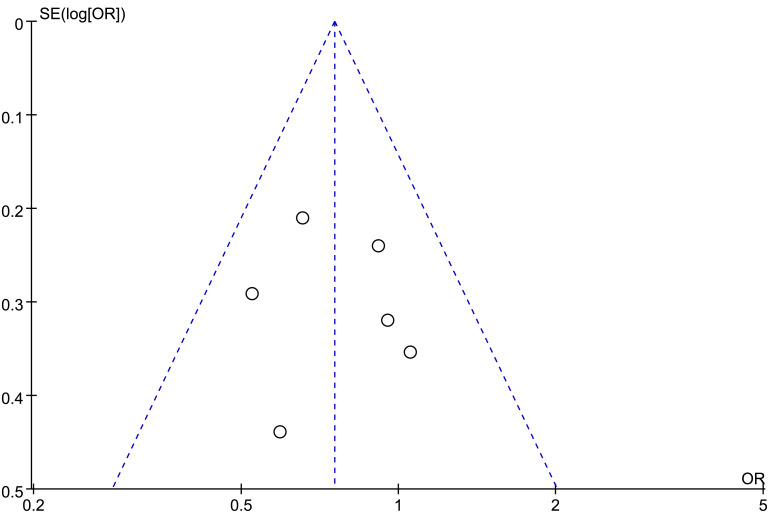
Funnel plot (assessment of publication bias).

Publication bias was assessed both graphically and descriptively. First, a risk of bias assessment plot (Fig. 6) was generated for the seven included studies using the Newcastle-Ottawa Scale domains to visualize potential biases in participant selection, comparability, and outcome assessment. Second, funnel plot (Fig. 7) analysis was performed to inspect for small-study effects and potential publication bias. Given the limited number of studies, a funnel plot was constructed for the analysis of sex (male *vs.* female), as this was a consistent and universally reported baseline characteristic across all studies. Funnel plot showed a roughly symmetrical distribution of study points around the pooled effect estimate, indicating no overt publication bias for this variable. However, this assessment is limited and unreliable due to the small number of included studies (*n* < 10).

## Discussion

Patient responses to cancer therapy are inconsistent, even with recent advances in surgery and multidisciplinary care. Underlying health status at baseline is a key determinant of this variability. Therefore, meticulous assessment of pre-treatment conditions is imperative. By integrating markers of systemic inflammation (CRP), nutritional status (albumin), and host immunity (lymphocyte count), the CALLY index provides a multidimensional assessment of the host’s pathophysiological state (Wu et al., 2025). Chronic inflammation, as reflected by an elevated CRP level, promotes tumor progression, angiogenesis, and metastasis; hypoalbuminemia indicates malnutrition and catabolic states detrimental to treatment tolerance and tissue repair (Linciano et al., 2024). Lymphopenia, a marker of impaired cell-mediated immunity, diminishes antitumor surveillance and response (Müller et al., 2021). The CALLY index’s superiority over single biomarkers likely stems from its ability to simultaneously capture these interconnected biological processes, offering a more holistic reflection of the host–tumor interaction than isolated parameters such as NLR, PLR, or mGPS (Fan et al., 2025).

Initially, the CALLY index was proposed as an immune–nutrition score for patients with hepatocellular carcinoma, a convenient and concise comprehensive indicator that is significantly correlated with the prognosis of patients with liver cancer (Iida et al., 2022). Continuous studies have shown that the CALLY index is associated with the prognosis of various cancers. A meta-analysis involving 7,951 patients with digestive system cancers revealed that a low pretreatment CALLY index is significantly associated with poor prognosis (Wu et al., 2025). Moreover, a recent large study of the National Health and Nutritional Examination Surveys from 1999 to 2018 in the USA reported that a low CALLY index is a significant predictor of all-cause mortality in various cancer subtypes (Zhu et al., 2024). These results collectively suggest that the CALLY index may serve as a useful integrative biomarker for risk stratification in EC.

This systematic review and meta-analysis represents the first comprehensive synthesis of evidence investigating the prognostic value of the CALLY index in patients with EC. By pooling 2,243 patients across seven contemporary cohort studies, we demonstrated that a high pretreatment CALLY index is significantly associated with improved survival outcomes, serving as an independent prognostic factor as revealed by the univariate and multivariate analyses. In particular, the patients with a high CALLY index exhibited superior 5-year OS (RR = 1.88, 95% CI [1.62–2.19]) and 5-year RFS (RR = 1.90, 95% CI [1.48–2.44]) than those with a low CALLY index. Furthermore, the CALLY index remained a significant independent predictor of OS (multivariate RR = 2.40) and RFS (multivariate RR = 1.99) after the adjustments for TNM stage, treatment modality, and other conventional risk factors. These results collectively affirmed the clinical utility of the CALLY index as a novel, integrative biomarker for risk stratification in EC.

Compared with those in the high CALLY index group, a high proportion of the patients with EC in the low CALLY index group had postoperative complications, anastomotic leakage, T stage greater than 1, lymph node metastasis, lymph-vascular invasion, and adjuvant chemotherapy (Li et al., 2025). Aoyama et al. (2024) analyzed the details of postoperative surgical complications in patients with EC and found a significant higher incidence of anastomotic leakage in the low CALLY index group compared with that in the high CALLY index group (40.2% *vs* 27.5%, *P* = 0.03). Fan et al. (2025) and Xu et al. (2025) demonstrated that a low CALLY index was significantly associated with increased postoperative pneumonia risk in patients with EC (*P* < 0.001). Jia et al. (2025) verified the superior predictive ability of the CALLY index by comparing its C-index and area under the curve against those of the glucose-to-lymphocyte ratio, Naples prognostic score, NLR, PLR, and SII. Their proposed nomogram prediction model—combining the CALLY index, smoking status, and TNM staging—showed better predictive performance than traditional TNM staging (Jia et al., 2025). Ma et al. (2024) found that among patients with EC receiving neoadjuvant chemotherapy, those with a low CALLY index had a worse prognosis than those with a high CALLY index. For patients with EC who have a TNM stage II–III and a serological CALLY index ≤2.55, Meng et al. (2025) recommend neoadjuvant therapy to reduce tumor burden and modulate the immunosuppressive microenvironment. Failure to elevate the CALLY index post-neoadjuvant therapy may indicate treatment refractoriness, warranting either prompt regimen modification or a shift to palliative care to avoid nonbeneficial interventions and optimize resource allocation.

Our analysis of clinical characteristics revealed that patients with a high CALLY index are likely to be male with early T status, low rates of vascular invasion, and early TNM stages. This profile aligns with the observed survival advantage, as these factors themselves are established indicators of less aggressive disease biology and good prognosis. The lack of significant differences in tumor differentiation, lymph node metastasis, neoadjuvant therapy rates, or postoperative complications between the high and low CALLY index groups may be due to the insufficient number of studies and data.

The lack of significant difference in neoadjuvant therapy rates between CALLY groups (OR = 1.45, *P* = 0.07). The trend (OR > 1) may indicate more frequent neoadjuvant therapy in the low-CALLY group, aligning with advanced disease (Ma et al., 2024; Aoyama et al., 2024). Non-significance may result from limited statistical power or protocol heterogeneity. Importantly, treatment decisions are guided by TNM stage—itself correlated with CALLY—rather than the index directly (Aoyama et al., 2024). Therefore, a low baseline CALLY reflects a pro-inflammatory, malnourished state associated with aggressive tumor biology and poor prognosis, independent of treatment received (Jia et al., 2025). Future studies should investigate dynamic CALLY changes during and after neoadjuvant therapy as a potential response and post-therapeutic risk stratification biomarker.

According to our meta-analysis and the existing research evidence, the CALLY index has several immediate clinical implications. First, pre-operative CALLY assessment can refine risk stratification within existing TNM stages, especially among patients scheduled for neoadjuvant chemoradiotherapy for whom TNM loses predictive accuracy. Second, the patients with a low CALLY index displayed high rates of postoperative complications, suggesting that targeted prehabilitation—encompassing nutritional optimization, exercise training, and inflammatory modulation—could translate into measurable peri-operative gains. Third, the dynamic changes in the CALLY index after neoadjuvant chemoradiotherapy emerged as a putative early-response biomarker. Finally, the simplicity and universal availability of the CALLY index from routine blood panels make it readily implementable without additional burden. Identifying patients with a low CALLY index could prompt intensified surveillance protocols, consideration of aggressive treatment strategies (*e.g.*, extended adjuvant therapy), or enrollment in trials exploring nutritional or immunomodulatory interventions aimed at improving the host’s systemic environment.

Our review has a number of strengths and limitations. The strengths include strict adherence to the PRISMA guidelines, prospective registration (PROSPERO:CRD420251125031), and comprehensive sensitivity analyses that confirmed the stability of our estimates. However, this study also has limitations that warrant consideration. First, only seven studies were included, and all of them were retrospective, raising the possibility of selection bias and residual confounding. Second, all the included studies originated from East Asia (Japan and China), where esophageal squamous cell carcinoma predominates. The generalizability of our findings to Western populations with high rates of esophageal adenocarcinoma requires further investigation, as tumor biology and host factors may differ. Third, the optimal CALLY index cut-off varied across the studies (range of 1.70–12.08), reflecting the absence of universal standardization and the use of different statistical methods (*e.g.*, ROC curve optimization, survival rate analysis, or distribution-based splits). Finally, publication bias assessment was limited by the small number of studies (*n* = 7). Although funnel plot analysis showed no overt bias, the risk of selective reporting cannot be excluded.

Beyond statistical heterogeneity, several clinical and methodological sources of diversity merit interpretive consideration. First, all included cohorts were East Asian, limiting generalizability to Western (as discussed above). Second, treatment approaches varied—including surgery alone, neoadjuvant chemotherapy, and neoadjuvant chemoradiotherapy—reflecting real-world practice. Notably, the uniformly low I^2^ values (<50%) for both OS and RFS analyses indicate that the prognostic effect of the CALLY index remains robust across these different therapeutic contexts, supporting its role as a baseline host-risk indicator independent of subsequent management. Third, the optimal CALLY cutoff ranged widely (1.70–12.08), driven by differences in cohort characteristics, outcome definitions, and statistical methods. Collectively, these considerations do not undermine the consistency of our pooled estimates but rather contextualize them, reinforcing the CALLY index as a promising yet still-evolving biomarker. Future research should focus on the following key areas: large-scale prospective validation of the CALLY index in patients receiving immune-checkpoint blockade, ideally within RCTs; investigation of the index’s dynamic changes during treatment and its correlation with therapeutic response; and exploration of the index’s utility in guiding specific interventions (*e.g.*, nutritional support, anti-inflammatory agents, or immunotherapy).

## Conclusion

The pretreatment CALLY index demonstrates robust and independent associations with OS and RFS in patients with EC. Its ability to integrate inflammation, nutrition, and immune status into a single metric offers a significant advantage over traditional single-parameter biomarkers. The CALLY index is readily calculable from routine blood tests, making it a practical and cost-effective tool for improving risk stratification beyond conventional TNM staging. Its integration into clinical practice has the potential to refine prognostication, guide therapeutic decision-making, identify high-risk patients warranting close surveillance or novel interventions, and ultimately contribute to improved patient outcomes. Prospective multicenter studies are warranted to confirm these findings and establish the role of the CALLY index in personalized EC management paradigms.

##  Supplemental Information

10.7717/peerj.21277/supp-1 Supplemental Information 1 Comparison of Sex

10.7717/peerj.21277/supp-2 Supplemental Information 2 Comparison of Differentiation

10.7717/peerj.21277/supp-3 Supplemental Information 3 Comparison of Neoadjuvant therapy

10.7717/peerj.21277/supp-4 Supplemental Information 4 Comparison of T status

10.7717/peerj.21277/supp-5 Supplemental Information 5 Comparison of Lymph node metastasis

10.7717/peerj.21277/supp-6 Supplemental Information 6 Comparison of Vessel invasion

10.7717/peerj.21277/supp-7 Supplemental Information 7 Comparison of TNM stage

10.7717/peerj.21277/supp-8 Supplemental Information 8 Comparison of Postoperative complication

10.7717/peerj.21277/supp-9 Supplemental Information 9 PRISMA checklist

10.7717/peerj.21277/supp-10 Supplemental Information 10 Raw data

## Additional Information and Declarations

### Data Availability

The following information was supplied regarding data availability: This is a Systematic review/Meta-analysis The raw data are available in the Supplemental File.
