# Supplementary figures and images for: Prognostic significance of the CRP–albumin–lymphocyte (CALLY) index in esophageal cancer: systematic review and meta-analysis
Source: PeerJ. 2026 May 22;14:e21277. doi: 10.7717/peerj.21277 (PMC13200621; doi:10.7717/peerj.21277)

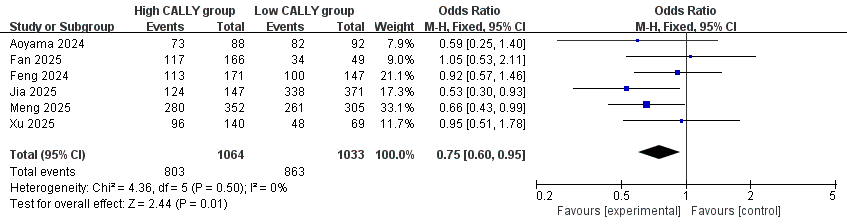

Supplement: Supplemental Information 1 [file peerj-14-21277-s001.png]

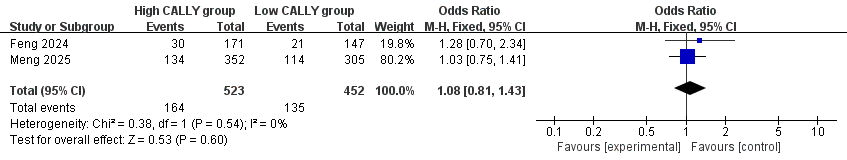

Supplement: Supplemental Information 2 [file peerj-14-21277-s002.png]

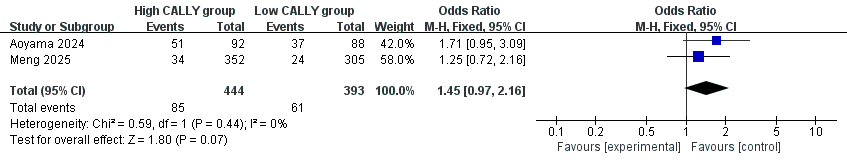

Supplement: Supplemental Information 3 [file peerj-14-21277-s003.png]

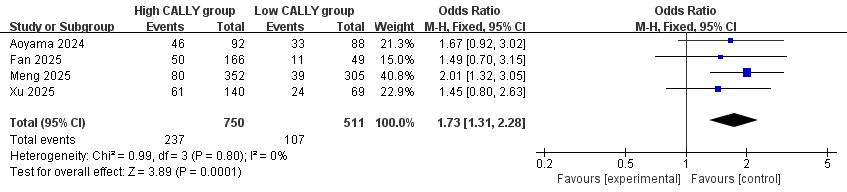

Supplement: Supplemental Information 4 [file peerj-14-21277-s004.png]

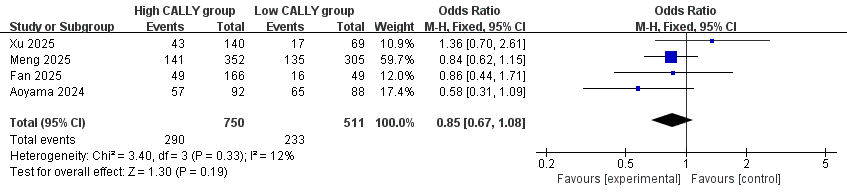

Supplement: Supplemental Information 5 [file peerj-14-21277-s005.png]

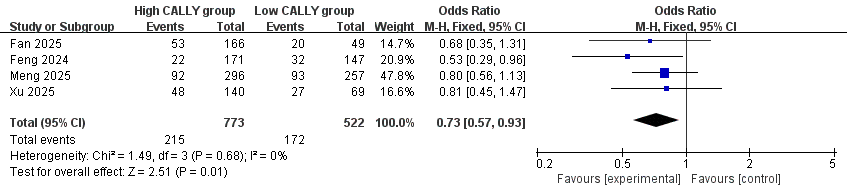

Supplement: Supplemental Information 6 [file peerj-14-21277-s006.png]

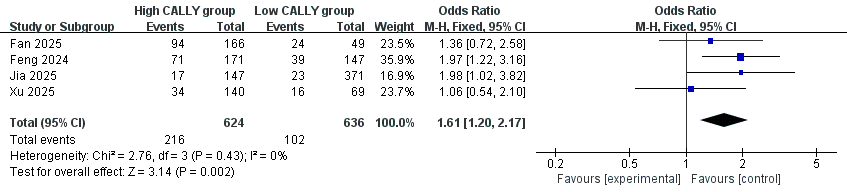

Supplement: Supplemental Information 7 [file peerj-14-21277-s007.png]

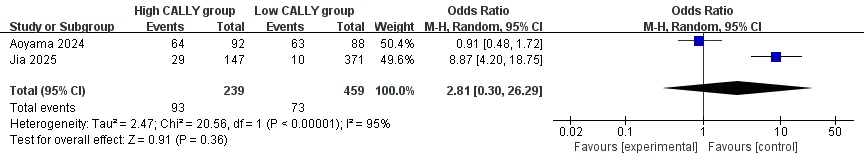

Supplement: Supplemental Information 8 [file peerj-14-21277-s008.png]
